# The Effect of IL-35 on the Expression of Nasal Epithelial-Derived Proinflammatory Cytokines

**DOI:** 10.1155/2021/1110671

**Published:** 2021-12-03

**Authors:** Mingrong Nie, Qingxiang Zeng, Luo Xi, Yiquan Tang, Renzhong Luo, Wenlong Liu

**Affiliations:** Department of Otolaryngology, Guangzhou Women and Children's Medical Center, Guangzhou Medical University, Guangzhou, China

## Abstract

**Background:**

Airway epithelium plays an important role during the development of allergic rhinitis (AR), which is characterized by production of thymic stromal lymphopoietin (TSLP), interleukin 33 (IL-33), and interleukin 25 (IL-25). IL-35, mainly expressed by Treg cells, have negative regulation in Th2, Th17, and ILC2 inflammation. However, the effect of IL-35 on human nasal epithelial cells (HNECs) especially the secretion of nasal epithelial-derived proinflammatory cytokines as well as the possible mechanism is still unclear.

**Methods:**

HNECs were cultured and stimulated by various stimulators. The expression of IL-33, IL-25, TSLP, eotaxin-1, eotaxin-2, and eotaxin-3 from supernatant was measured using real-time reverse transcription-polymerase chain reaction (RT-PCR) and enzyme-linked immunosorbent assay (ELISA). AR mice were developed to verify the effect of IL-35 on nasal epithelial cells in vivo.

**Results:**

After Poly I:C stimulation, IL-35 inhibited the production of IL-25, and TSLP from HNECs increased significantly compared with baseline levels (*P* < 0.05). After *Dermatophagoides pteronyssinus* or *Aspergillus fumigatus* stimulation, IL-35 inhibited the production of IL-25, IL-33, and TSLP from HNECs increased significantly compared with baseline levels (*P* < 0.05). After *Dermatophagoides pteronyssinus*, IL-35 inhibited the production of eotaxin-1, eotaxin-2, and eotaxin-3 released from HNECs increased significantly compared with baseline levels (*P* < 0.05). Similarly, IL-35-treated AR mice presented with decreased expression of IL-33, IL-25, TSLP, eotaxin-1, eotaxin-2, and eotaxin-3 in nasal lavage fluid.

**Conclusion:**

IL-35 suppressed both type 2 inflammation-inducing cytokines and eosinophil chemotactic factor from HNECs, suggesting the important role of IL-35 during the development of AR.

## 1. Introduction

Allergic rhinitis (AR), one of the most common diseases of the upper airway, affects more and more adults and children with increasing worldwide prevalence [1]. Classically, type 2 CD4+ T-helper (Th2) is believed to play important roles during the development of AR. Activated Th2 cells produce type 2 cytokines, including interleukin- (IL-) 4, IL-5, and IL-13, which promote the infiltration of mucosal eosinophils [2]. Group II innate lymphoid cells (ILC2), newly identified cells, can also produce IL-13, IL-5, IL-4, and IL-9 after activation by epithelial-derived cytokines even in the absence of T cells [3].

Airway epithelium acts as both physical barrier and immunologically active interface between environmental stimuli and respiratory system in airway inflammation. After interaction with different environmental stimuli, airway epithelium can secrete thymic stromal lymphopoietin (TSLP), interleukin 33 (IL-33), and interleukin 25 (IL-25), which contribute to the initiation of type 2 and eosinophil inflammation [4].

IL-35, which belongs to the IL-12 cytokine family, consists of Epstein-Barr virus-induced gene 3 (EBI3) chain and IL-12p35 chain [5]. The receptors of IL-35 included IL-12R*β*2 and gp130 or a heterodimer of IL-12R*β*2:gp130 [6]. IL-35 is mainly expressed by Treg cells and in turn induces proliferation of Treg cells [7]. IL-35 inhibits the proliferation and function of Th2 and Th17 cell populations in vitro [8, 9]. Moreover, our previous study found that IL-35 inhibited ILC2 responses directly or through mutual contact between IL-35-induced Treg and ILC2 in AR [10]. However, few studies explored the regulation of IL-35 on epithelial-derived cytokines.

In this study, we aimed to investigate the effect of IL-35 on human nasal epithelial cells (HNECs), especially the secretion of nasal epithelial-derived proinflammatory cytokines as well as the possible mechanism.

## 2. Methods

### 2.1. Cell Culture and Treatment

Human nasal epithelial cells (HNEpC, PromoCell, Germany) were thawed and cultured in Airway Epithelial Cell Growth Medium (PromoCell, Germany) at 37°C with 5% CO_2_. The cells were subcultured according to the instructions when they reached 70-90% confluency with density of 10000 cells per cm^2^.

The confluent cells were stimulated by various stimulators, which included 10-200 ng/mL IL-35, 25 *μ*g/mL Poly I:C, 1.6 *μ*g/mL of Dermatophagoides pteronyssinus, 2.6 *μ*g/mL of Aspergillus fumigatus, 10 ng/mL of TNF-*α*, and 0.1 ng/mL of IL-1*β*. The HNECs were cultured with different stimulator combinations for 24 hours.

### 2.2. Animal Model

Thirty male BALB/c mice (eight-week-old) were obtained (Guangdong animal experiment center, China) and raised in pathogen-free environment. The sensitization was performed intraperitoneally using 100 *μ*g of Der p1 and 1.6 mg Al(OH)_3_ mixed with 100 *μ*L of PBS on days 1, 5, 14, and 21. Allergic inflammation was induced by providing 100 *μ*g of Der p1, IL-35 (10-200 ng/mL), or anti-IL-35 (100 ng/mL) in 100 *μ*L of PBS nasally from day 22 to 28. The nasal lavage fluid (NLF) was collected by nasal cavity perfusion using a catheter from back to front with 1.1 mL of PBS. Nasal mucosa tissues were also collected for further experiments. All animal care and experimental protocols were approved by local ethics committee boards.

### 2.3. Real-Time Reverse Transcription-Polymerase Chain Reaction (RT-PCR)

Total RNA was isolated from HNECs or nasal tissue using the TRIzol reagent (Invitrogen) according to the manufacturer's instructions. The cDNA was synthesized from two micrograms of total RNA using an oligo (dT)18 primer by the Superscript First Synthesis System for RT-PCR (Life Technologies, Carlsbad, CA). Real-time polymerase chain reaction was done using an ABI PRISM 7300 Detection System (Applied Biosystems, Foster City, CA, USA). The primers were as follows: IL-33 forward, 5′-GACTCCTCCGAACACAGAGC-3′, IL-33 reverse, 5′-CCCAGCTTGAAACACAAGGC-3′; IL-25 forward, 5′-CCTGGAGATATGAGTTGGACAGAGA-3′, IL-25 reverse, 5′-CCATGTGGGAGCCTGTCTGTA-3′; TSLP forward, 5′-TGTAGCAATCGGCCACATTG-3′, TSLP reverse, 5′-CAGCCTTAGTTTTCATGGCGA-3′; eotaxin-1 forward, 5′-CCTCTCACGCCAAAGCTCACACCTTC-3′, eotaxin-1 reverse, 5′-CGGCACAGATATCCTTGGCCAGTTTG-3′; eotaxin-2 forward, 5′-CCTTCTGTTCCTTGGTGTCTGTG-3′, eotaxin-2 reverse, 5′-TTCATGTACCTCTGGACCCACTC-3′; eotaxin-3 forward, 5′-GGAACTGCC-ACACGTGGGAGTGAC-3′, eotaxin-3 reverse, 5′-CTCTGGGAGGAAACACCCTCTCC-3′; SOCS3 forward: 5′-CACCTTCTTGGTGCGCG-3′, SOCS3 reverse: 5′-AAGCCATCTTCACGCTGAGCTCTG-3′; STAT3 forward: 5′-AATATAGCCGATTCCTGCAAGAG-3′, STAT3 reverse: 5′-TGGCTTCTCAAGATACCTGCTC-3′; and *β*-actin forward, 5′-AAG ATG ACC CAG ATC ATG TTT GAG ACC-3′ and *β*-actin reverse, 5′-AGC CAG GTC CAG ACG CAG GAT-3′. For determination of relative quantitation of gene expression, we used the 2^-*ΔΔ*Ct^ method, with *β*-actin as the internal control for normalization.

### 2.4. Enzyme-Linked Immunosorbent Assay (ELISA)

The levels of IL-33 (1.51 pg/mL), IL-25 (11.7 pg/mL), TSLP (9.87 pg/mL), eotaxin-1 (5 pg/mL), eotaxin-2 (14.3 pg/mL), and eotaxin-3 (0.125 pg/mL) were detected using commercial available ELISA kits (R&D Systems, Minneapolis, MN, USA) according to the manufacturer's protocols.

### 2.5. Statistical Analysis

Values are presented as the mean ± SD except additional note. Comparisons were performed by one-way ANOVA with Fisher's protected least significant difference test for comparison among three or more groups. *P* < 0.05 was considered significantly different. All analyses were performed using statistical software SPSS (version 18).

## 3. Results

### 3.1. Effect of IL-35 on Proinflammatory Cytokines Produced by HNECs

**Figure 1:**
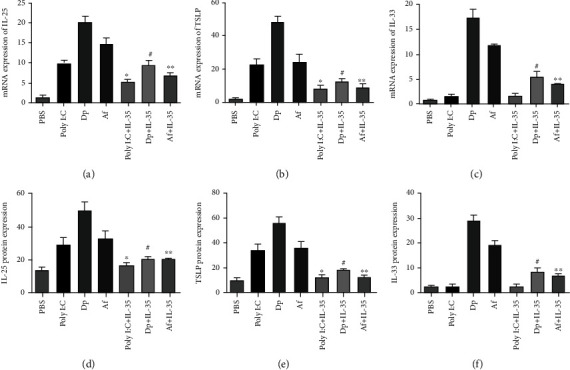
The expression of IL-25, IL-33, and TSLP by human nasal epithelial cells after various stimulators. (a–c) The mRNA expression of IL-25, IL-33, and TSLP by human nasal epithelial cells. (d–f) The protein expression of IL-25, IL-33, and TSLP by human nasal epithelial cells. Poly I:C: 25 *μ*g/mL polyinosinic-polycytidylic acid; Dp: 1.6 *μ*g/mL of Dermatophagoides pteronyssinus; Af: 2.6 *μ*g/mL of Aspergillus fumigatus; IL-35: 100 ng/mL interleukin-35. ^∗^Compared with Poly I:C, *P* < 0.05; ^#^compared with Dp, *P* < 0.05; ^∗∗^compared with Af, *P* < 0.05. Three independent tests were performed for every experiment.

After Poly I:C stimulation, IL-25 and TSLP released from HNECs increased significantly compared with baseline levels. When IL-35 was added, the IL-25 and TSLP production was inhibited significantly (Figure 1). The expression of IL-33 was not changed after Poly I:C or IL-35 stimulation (Figure 1).

After *Dermatophagoides pteronyssinus* stimulation, IL-25, IL-33, and TSLP released from HNECs increased significantly compared with baseline levels. When IL-35 was added, the IL-25, IL-33, and TSLP production was inhibited significantly (Figure 1).

After *Aspergillus fumigatus* stimulation, IL-25, IL-33, and TSLP released from HNECs increased significantly compared with baseline levels. When IL-35 was added, the IL-25, IL-33, and TSLP production was inhibited significantly (Figure 1).

### 3.2. Effect of IL-35 on Eosinophil Chemotactic Factors Produced by HNECs

**Figure 2:**
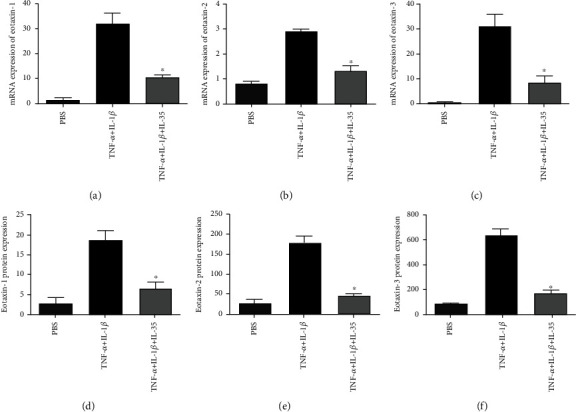
The expression of eotaxins by human nasal epithelial cells after various stimulators. (a–c) The mRNA expression of eotaxin-1, eotaxin-2, and eotaxin-3 by human nasal epithelial cells. (d–f) The protein expression of eotaxin-1, eotaxin-2, and eotaxin-3 by human nasal epithelial cells. Stimulators included 10 ng/mL of TNF-*α*, 0.1 ng/mL of IL-1*β*, and 100 ng/mL interleukin-35. ^∗^Compared with TNF-*α*+IL-1*β*, *P* < 0.05. Three independent tests were performed for every experiment.

After TNF-*α* and IL-1*β* stimulation, eotaxin-1, eotaxin-2, and eotaxin-3 released from HNECs increased significantly compared with baseline levels. When IL-35 was added, the eotaxin-1, eotaxin-2, and eotaxin-3 production was inhibited significantly (Figure 2).

### 3.3. Effect of IL-35 on Signaling Pathways of HNECs

**Figure 3:**
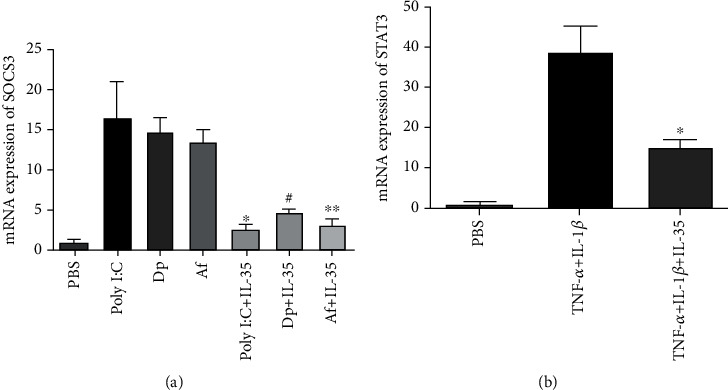
The expression of SOCS3 and STAT3 by human nasal epithelial cells after various stimulators. (a) The mRNA expression of SOCS3 by human nasal epithelial cells. Poly I:C: 25 *μ*g/mL polyinosinic-polycytidylic acid; Dp: 1.6 *μ*g/mL of Dermatophagoides pteronyssinus; Af: 2.6 *μ*g/mL of Aspergillus fumigatus; IL-35: 100 ng/mL interleukin-35. ^∗^Compared with Poly I:C, *P* < 0.05; ^#^compared with Dp, *P* < 0.05; ^∗∗^compared with Af, *P* < 0.05. (b) The mRNA expression of STAT3 by human nasal epithelial cells. Stimulators included 10 ng/mL of TNF-*α*, 0.1 ng/mL of IL-1*β*, and 100 ng/mL interleukin-35. ^∗^Compared with TNF-*α*+IL-1*β*, *P* < 0.05. Three independent tests were performed for every experiment.

IL-35 inhibited SOCS3 expression from HNECs significantly after Poly I:C, *Dermatophagoides pteronyssinus*, or *Aspergillus fumigatus* stimulation. IL-35 inhibited the expression of STAT3 from HNECs stimulated with TNF-*α* and IL-1*β* (Figure 3).

### 3.4. Effect of IL-35 of HNECs on Allergic Mice

**Figure 4:**
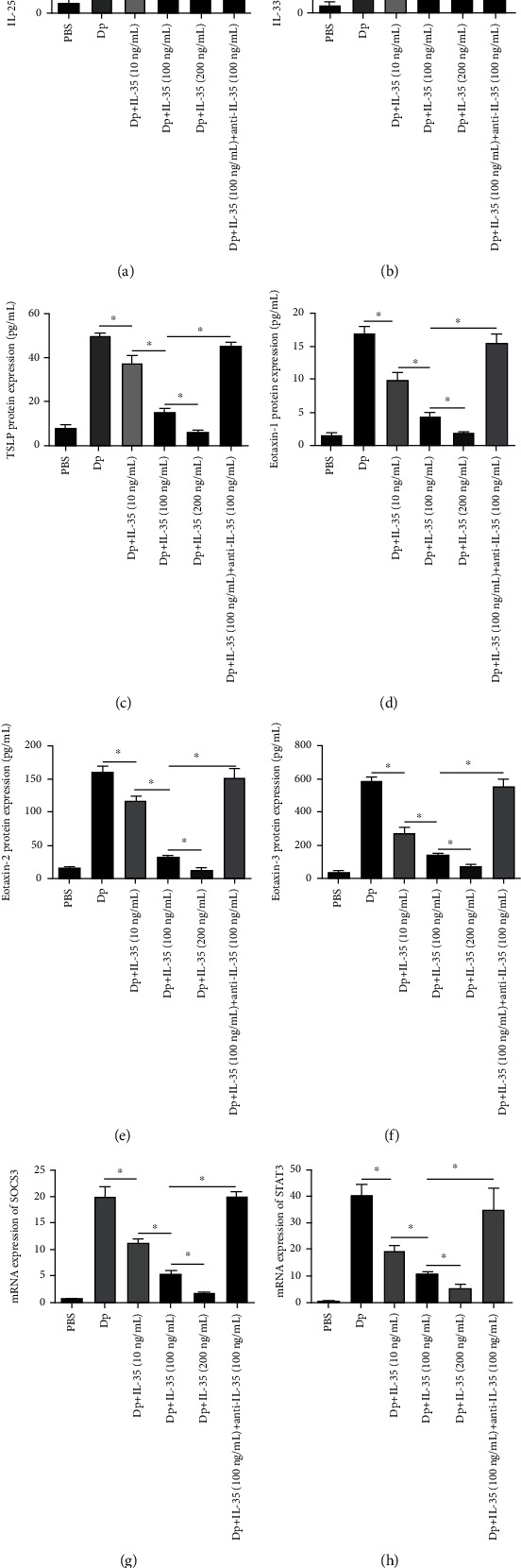
Effect of IL-35 of HNECs in allergic mice. (a–c) The protein expression of IL-25, IL-33, and TSLP by nasal lavage fluid in allergic mice. (d–f) The protein expression of eotaxin-1, eotaxin-2, and eotaxin-3 by nasal lavage fluid in allergic mice. (g, h) The mRNA expression of SOCS3 and STAT3 by nasal mucosa tissue in allergic mice. ^∗^Compared between groups, *P* < 0.05. Three independent tests were performed for every experiment.

IL-35-treated AR mice presented with decreased expression of IL-33, IL-25, TSLP, eotaxin-1, eotaxin-2, and eotaxin-3 in nasal lavage fluid in a dose-dependent manner, whereas anti-IL-35 reversed the effect of IL-35 (Figure 4). Moreover, the mRNA expression SOCS3 and STAT3 by nasal mucosa tissue was also inhibited by IL-35 in a dose-dependent manner (Figure 4).

## 4. Discussion

In the present study, we found that IL-35 suppressed both type 2 inflammation-inducing cytokines and eosinophil chemotactic factors from HNECs, suggesting the important role of IL-35 during the development of AR.

IL-35, a recently identified cytokine, can inhibit the production of IL-4, IL-5, and IL-13 as well as IgE from bronchoalveolar lavage fluid (BALF) of the allergic mouse model [11]. Moreover, intraperitoneal injection of IL-35 can reduce the number of eosinophils in BALF [12]. These results suggested that IL-35 had a potent inhibitive role for type 2 inflammation and eosinophil infiltration. However, its mechanism was not clear.

TSLP, IL-33, and IL-25 secreted from HNECs were known as “master switches” in the development of allergic diseases. Of which, TSLP induces both Th2 and ILC2 [13]. IL-25 promotes the production of type 2 cytokines IL-5 and IL-13 from Th2 and ILC2 [14, 15]. IL-33 can activate eosinophils directly except for inducing type 2 cytokines from Th2 and ILC2 [16, 17]. Therefore, we suppose that IL-35 may be involved in allergic diseases by regulating the expression of TSLP, IL-33, and IL-25 from HNECs.

As we expected, our results found that IL-35 can inhibit the production of IL-25, IL-33, and TSLP from HNECs induced by *Dermatophagoides pteronyssinus* and *Aspergillus fumigatus*, suggesting that IL-35 posed a negative impact on allergic inflammation in the early stage. Similarly, IL-35 decreased the expression of IL-25 and TSLP from HNECs after Poly I:C stimulation. Interestingly, we found that the expression of IL-33 was not changed after Poly I:C, which may be attributed to the activation of different Toll-like receptors. Similarly, Boita et al.'s study found no change in IL-33 expression from epithelial cells derived from nasal polyps following Poly I:C stimulation [18].

The airway epithelium is also one of the main sources of chemoattractant for eosinophils, which included eotaxin-1, eotaxin-2, and eotaxin-3 [19]. Our results also confirmed that IL-35 inhibited the expression of eotaxins from HNECs significantly. Consistently, Kanai et al.'s study also demonstrated that IL-35 suppresses LPS-induced airway eosinophilia by reducing local production of eotaxin-1 and eotaxin-2 [20].

SOCS3 is involved in Th2 differentiation, while STAT3 was involved in eotaxin expression [20, 21]. Therefore, we detected the changes of these signaling pathways during IL-35 treatment. Our data showed that decreased SOCS3 and STAT3 expression from HNECs was correlated with the release of proinflammatory cytokines and chemoattractant for eosinophils, which is consistent with Kanai et al.'s reports [20].

Consistently, our in vivo study using the AR mouse model also showed a similar effect of IL-35 with cell model experiments. However, the expression of IL-35 in the body may be affected by multiple pathways. The detailed mechanism in the pathophysiological process needed further exploration.

In sum, IL-35 inhibited the expression of IL-25, IL-33, TSLP, and eotaxins from HNECs and regulated Th2, ILC2, and eosinophil inflammation in the early stage. IL-35 may be a potential target in the future treatment of AR.

## Data Availability

The datasets used and/or analyzed during the current study are available from the corresponding author on reasonable request.
